# Gender- and age-stratified analyses of ADHD medication use in children and adolescents in Finland using population-based longitudinal data, 2008–2018

**DOI:** 10.1177/1403494820901426

**Published:** 2020-01-27

**Authors:** Miika Vuori, Anna Koski-Pirilä, Jaana E. Martikainen, Leena Saastamoinen

**Affiliations:** 1 Department of Teacher Education, Turku Institute for Advanced Studies, University of Turku, Finland; 2 Research Unit, Social Insurance Institution of Finland, Helsinki, Finland (Kela); 3 Analytics Unit, Social Insurance Institution of Finland, Helsinki, Finland (Kela)

**Keywords:** Attention-deficit hyperactivity disorder, central stimulants, prevalence, children, adolescents, register-based population study

## Abstract

**Aims:** This study examined medication use for attention-deficit/hyperactivity disorder (ADHD) among children and adolescents by gender in Finland during 2008–2018. **Methods:** Aggregated data on medication use for ADHD from 2008 to 2018 were extracted from the nationwide register on reimbursed prescriptions. The annual prevalence of ADHD medication use was calculated as the number of children (6–12 years) and adolescents (13–17 years) per calendar year with at least one ADHD medication purchase divided by the number of children and adolescents in the population. Population prevalence for children was also examined by birth month. **Results:** In 2008, the prevalence rates for males were 1.26% in children and 0.93% in adolescents, and for females 0.21% and 0.14%, respectively. In 2018, the prevalence rates for males were 4.42% in children and 4.21% in adolescents, and for females 0.99% and 1.28%, respectively. Male-to-female ratios decreased during the study period from 6.0:1 to 4.5:1 (children) and from 6.6:1 to 3.3:1 (adolescents). ADHD medication use was more common among males and females (aged 6–12 years) born in May–August or September–December than among males and females born in January–April. **Conclusions: The prevalence of ADHD medication use has continued to increase in Finland. Although use has increased more rapidly among females resulting in lower male-to-female ratios, medication use among females is considerably lower compared with males. Consequently, gender discrepancy in 2018 was relatively large, particularly among children. Future studies should also consider reporting annual prevalence by children’s birth month.**

## Introduction

Attention-deficit/hyperactivity disorder (ADHD) is a neurodevelopmental disorder characterized by behavioural symptoms of inattention, hyperactivity, and impulsivity. ADHD is further associated with considerable social functioning deficits [1–3]. Pooled prevalence estimates for ADHD in population aged 6–17 ranges from 3.4% [4] to 7.2% [5]. Although the rates of children with clinically diagnosed ADHD have increased [2, 6–9], there is no evidence of an increase in clinically significant ADHD-like traits at the extreme end of the distribution in the population [10–11]. Male-to-female ratios of ADHD diagnoses in community and clinical samples range from 2.4:1 to 4.0:1 [2,6].

Pharmacotherapy is an important part of ADHD treatment [2]. Stimulants and non-stimulants are superior to placebo in treating ADHD core symptoms and ADHD-related emotion dysregulation in the short term [12]. Parents and physicians are nowadays more likely to consider an ADHD diagnosis, and pharmacotherapy has become more acceptable [13]. ADHD medication use has markedly increased in many countries, and peaks in 9–14-year-old males [14–16]. In the Nordic countries, ADHD medication use among school-aged children has been most common in Iceland and least common in Finland [15–16]. Medication use has increased in Iceland, Sweden and Finland, but remained relatively stable in Norway and Denmark since 2010. However, trends beyond 2013 are not known [14–16].

Recent findings indicate that male-to-female ratios in medication use among child and youth population range from 2:1 to 6:1 [2,16], with the lowest gender discrepancies in Australia and the United States and the highest in Finland, Hong Kong and the UK [16]. Decreased gender discrepancy may reflect increased awareness of ADHD in females [17–19]. ADHD medication use in children and adolescents may also relate to differences in ADHD medication adherence [19–22] and gender-based effects of ADHD heterogeneity over the life course [2, 23–24]. Importantly, there is also a growing literature indicating that younger relative age within the school year is associated with increased ADHD medication use, particularly among the child population [25–27].

This paper extends the findings from recent Nordic and international comparison studies [14, 15] by establishing age- and gender-specific time trends (2008–2018) for ADHD medication use among Finnish children (aged 6–12 years) and adolescents (aged 13–17 years). In addition, prior studies have not examined annual prevalence by children’s birth month. The main hypotheses were that (a) ADHD medication use has increased in both genders, (b) the male-to-female ratio has decreased, and (c) and there is a small relative age effect in ADHD medication use in children.

## Methods

Data on ADHD medication purchases reimbursed by the National Health Insurance in 2008–2018 were extracted from the Finnish Prescription Register for children and adolescents aged 6–17. Use of ADHD medication was defined as at least one purchase of methylphenidate, atomoxetine, dexamphetamine, or lisdexamfetamine per calendar year. The annual prevalence of ADHD medication use was calculated as the number of children and adolescents with at least one reimbursed purchase of ADHD medication during the calendar year divided by the number of children and adolescents in the total population at the end of the year. The population was stratified by sex into two age groups: children (6–12 years) and adolescents (13–17 years). In Finland, the cut-off date for school eligibility is December 31. Thus, ADHD medication use in children by birth month was examined as follows: (a) born between January and April, (b) born between May and August, (c) born between September and December. According to national regulations, the study required no ethical approval.

## Results

**Figure 1.**
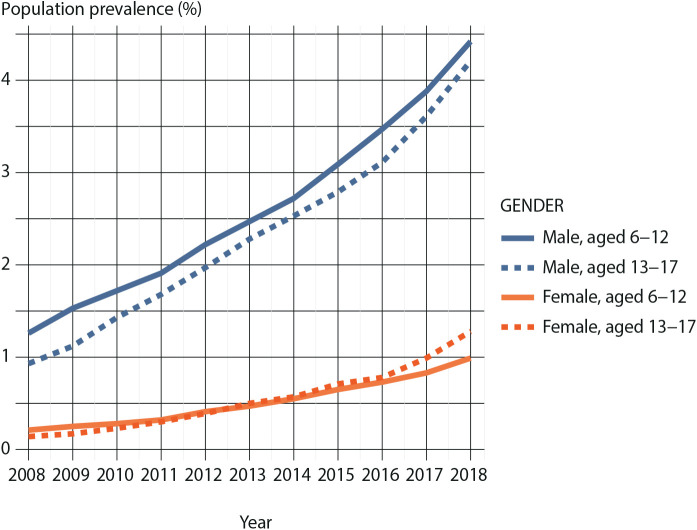
Annual prevalence of attention-deficit hyperactivity disorder medication use in children (aged 6–12 years) and adolescents (aged 13–17 years) by gender.

In 2008, the prevalence of ADHD medication use in the child population was 1.26% among males and 0.21% among females. During the study period, the prevalence increased steadily for both sexes. By 2018, the prevalence had increased to 4.42% among males, and to 0.99% among females (Figure 1).

In the adolescent population, the prevalence of ADHD medication in 2008 was 0.93% among males and 0.14% among females, but had increased to 4.21% among males and to 1.28% among females by 2018. Among males, ADHD medication use was more common in children than in adolescents throughout the study period, whereas among females, medication use has been slightly more common in adolescents than in children since 2013.

**Figure 2.**
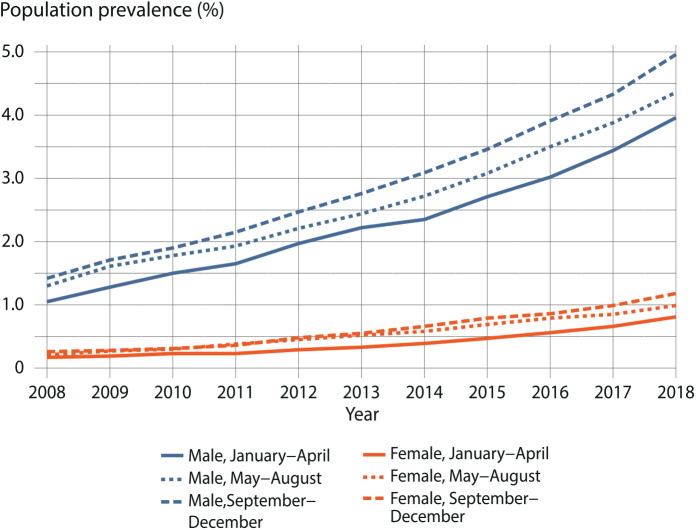
Annual prevalence of attention-deficit hyperactivity disorder medication use in children aged 6–12 years by gender and birth month.

In children, ADHD medication use was associated with birth month across genders. Data further showed that ADHD medication use was more common among males born later in the calendar year (i.e. May–August, September–December) when compared with their peers born in January–April across the whole study period. Among females, birth month and ADHD medication use were similarly associated (Figure 2).

Finally, among children receiving ADHD medication, the male-to-female ratio decreased during the study period from 6.0:1 to 4.5:1 (Table I), but the number of females on medication in 2018 did not reach the number of males on medication in 2008. In addition, gender discrepancy was also somewhat lower among children born in the last 3 months of the year than among those born in January–April. Among adolescents, the male-to-female ratio decreased more rapidly than in children from 6.6:1 to 3.3:1.

**Table I.** Descriptive statistics for gender differences in attention-deficit hyperactivity disorder medication use among children (aged 6–12 years) and adolescents (aged 13–17 years) in Finland.

Group	Year	Males		Females		M:F ratio
		*n*	%	*n*	%	
**Children**						
	2008	2629	1.26	422	0.21	6.0:1
	2018	9823	4.42	2105	0.99	4.5:1
	2018 vs. 2008	OR = 3.5 (3.4–3.7)		OR = 4.7 (4.3–5.3)		
**Children by birth month**						
Jan–Apr	2008	734	1.05	114	0.17	6.2:1
	2018	2917	3.96	567	0.81	4.9:1
	2018 vs. 2008	OR = 3.9 (3.6–4.2)		OR = 4.8 (3.9–5.8)		
May–Aug	2008	947	1.30	145	0.21	6.2:1
	2018	3346	4.36	721	0.99	4.4:1
	2018 vs. 2008	OR = 3.5 (3.2–3.7)		OR = 4.8 (4.0–5.7)		
Sep–Dec	2008	946	1.42	163	0.26	5.5:1
	2018	3560	4.96	817	1.18	4.2:1
	2018 vs. 2008	OR = 3.6 (3.4–3.9)		OR = 4.7 (3.9–5.5)		
**Adolescents**						
	2008	1573	0.93	230	0.14	6.6:1
	2018	6385	4.21	1854	1.28	3.3:1
	2018 vs. 2008	OR = 4.7 (4.4–4.9)		OR = 9.1 (7.9–10.5)		

Note: *n* = cases with medication, % = population prevalence.

M:F ratio = male-to-female ratio.

Crude odds ratios (OR) for comparing ADHD medication use between 2008 and 2018 within gender groups (95% confidence intervals in brackets).

## Discussion

From 2008 to 2018, the prevalence of ADHD medication use among Finnish children and adolescents increased steadily. This probably stems from a greater awareness of ADHD and changes in diagnostic and treatment practices [2,12,16]. For example, since 2012 the Finnish guideline for the treatment of ADHD has recommended that medication be considered for children aged 6 years and over if their ADHD symptoms are causing persistent significant impairment.

Comparing the results to other Nordic countries is somewhat difficult, as the trends beyond 2013 are unknown [14–16]. However, in Norway and Denmark, medication use did not increase between 2010 and 2013 [15–16]. If this trend has remained unchanged, the prevalence of ADHD medication use in males in Finland has reached the prevalence of Norway and Denmark. Moreover, when compared with findings of ADHD medication use across different world regions [25], our findings indicate that prescribing rates in the Finnish male population can no longer be considered low. Among females, however, medication use in Finland has remained relatively low compared with other countries [15–16].

Our study agreed with a recent population study from 13 countries and showed that the gender discrepancy in ADHD medication use is relatively large in Finland [16]. We observed male-to-female ratios of 4.5:1 for children in 2018. In comparison, male-to-female ratios in Denmark, Germany and the Netherlands in 2012 were 2.7:1, 3.6:1, and 3.0:1, respectively [14]. It seems that in Finnish healthcare settings ADHD may go unnoticed in the female population [17]. Prior studies further suggest that although clinic-referred females display similar levels of symptoms compared with males, the female gender is, on average, related to lower ratings on core symptoms of ADHD, which reflects a general delay in recognizing ADHD among females [2, 23–24].

Our study agreed with prior studies indicating that younger relative age within the school year is associated with increased ADHD medication use [25–27]. Although the observed association is somewhat modest across studies [25], it is important to acknowledge the late birthdate effect in diagnostic practices [27], but also in studies when reporting the annual prevalence of ADHD medication use. To our knowledge, this is the first study that has produced annual prevalence by birth month in more detail.

The results revealed that gender discrepancy has decreased more rapidly among adolescents than among children, and that among male prevalence rates for adolescents were lower relative to children across the whole study period. However, among females, prevalence has been slightly higher for adolescents since 2013. Prior studies show that the female gender may relate to increased adherence to pharmaceutical treatment in adolescence [20–21] and that ADHD medication use in adolescence is perhaps further influenced by co-occurring mental health problems [19–20] and the successfulness of the transition process from child to adolescent services [2,21–22]. However, these important issues remain beyond the scope of our study.

The major strength of our study is that the Prescription Register covers the whole Finnish population. The major limitation is that register data are based on claims data from pharmacies and it is unknown whether the medication is actually used.

Importantly, due to the increase in ADHD medication use there is a need for prospective population-based studies that examine the effectiveness and safety of pharmacotherapy, availability and adherence to the multimodal treatment components of ADHD, as well as the transition processes in children and adolescents in more detail.
